# Chromatin, Epigenetics and Plant Physiology

**DOI:** 10.3390/ijms21082763

**Published:** 2020-04-16

**Authors:** Miloslava Fojtová, Jiří Fajkus

**Affiliations:** 1 Mendel Centre for Plant Genomics and Proteomics, CEITEC, Masaryk University, CZ-62500 Brno, Czech Republic; fojtova@sci.muni.cz; 2 Laboratory of Functional Genomics and Proteomics, NCBR, Faculty of Science, Masaryk University, CZ-61137 Brno, Czech Republic; 3 Department of Cell Biology and Radiobiology, Institute of Biophysics of the Czech Academy of Sciences, v.v.i., CZ-61265 Brno, Czech Republic

The ever-increasing interest in epigenetics comes from the fact that in the diverse life situations of organisms, e.g., in cell differentiation, developmental decisions, or responses to biotic and abiotic stresses, it is primarily the reprogramming of the regulation of the existing genetic information, rather than its direct change, that solves the problem. Epigenetic mechanisms allow the organism to channel the appropriate response through diverse particular molecular tools modifying distinct levels of the structure of chromatin. Chromatin is thus marked with certain signals, for example, DNA methylation, posttranslational modifications of histones, incorporation of specific histone variants, or chromatin remodeling. These signals, written by respective enzymes and complexes, termed as epigenetic writers (e.g., DNA methyltransferases, histone methyltransferases, and histone acetyltransferases) have to find their readers—biomolecules recognizing the specific mark, and erasers which are capable of resetting the program. Recent data suggest a deep interconnection of individual epigenetic players, which frequently act together as components of the same multi-subunit complexes. For example, methylcytosine binding protein MeCP2 (a reader) recruits histone deacetylase (an eraser) and H3K9 histone methyltransferase Suv39h1 (a writer), and in this way reinforces the repressive state of a chromatin region [1,2]. Recent research in plants brings many novel findings elucidating the interdependence of diverse epigenetic mechanisms and their crosstalk with various signaling pathways, including the action of phytohormones and reactive oxygen and nitrogen species. Using these molecular tools, chromatin structure decides which particular set of genes will be active in a particular physiological process.

The special issue “Chromatin, Epigenetics and Plant Physiology” in the International Journal of Molecular Sciences comprises two review articles and eight original research papers (Table 1). All contributions deal with important aspects of epigenetic regulations of crucial cellular processes involved in plant growth and development.

Four articles, one review, and three research papers deal with chromatin remodeling complexes. Wang et al. [3] provide a review on the role of Arabidopsis SWR1 and INO80 chromatin remodeling complexes involved in the regulation of the replacement of nucleosomal H2A/H2B dimers with H2A.Z/H2B. The authors describe the composition of the SWR1/INO80-c complex and discuss its diverse nuclear roles ranging from repair processes to regulation of gene expression. Guo et al. [4] report on the involvement of the chromatin remodeler encoded by the *OsCHR4* gene in regulation of rice leaf morphology, via modulation of accumulation of cuticle wax on leaf surfaces and auxin biosynthesis. Expression profiles and subcellular localizations of tomato SWI3-like proteins were studied by Zhao et al. [5]. The authors further identify interactions of these subunits of the chromatin remodeling complex with proteins participating in the reproductive development. Their observations support the idea of evolutionary conservation of SWI3 physiological functions in different plant species. Similarly, the involvement of the SWI subunits of the chromatin remodeling complex in temperature-dependent regulation of plant growth and developmental responses in Arabidopsis is reported by Gratkowska-Zmuda et al. [6]. Altogether, these results demonstrate the importance of the proper chromatin remodeling in crucial cellular processes, including gene expression, cell cycle regulation, DNA replication and repair, and hormone signaling.

Results presented in two papers within the special issue “Chromatin, Epigenetics, and Plant Physiology” were obtained using specific advanced methodical approaches. Circular chromosome conformation capture (4C)-based method was utilized by Krispil et al. [7] for the detection of the entire scope of T-DNA insertions, by capturing the local enrichment of spatial chromosomal associations in their genomic proximity without prior knowledge of their genomic locations in Arabidopsis transgenic lines. This approach enables the identification of previously unmapped T-DNA insertions and related chromosomal rearrangements and is applicable to any plant with a sequenced genome. Lochmanová et al. [8] studied the acetylation of histone proteins by a mass spectrometry-based proteomic approach. They conclude that the effect of epigenetically active drugs on early plant development is complex and is not restricted to the ability of these compounds to modulate the levels of histone acetylation marks.

Koláčková et al. [9] solved an interesting problem of the spatial organization of parental genomes in the somatic nuclei of interspecific plant hybrids. They demonstrate that domains of introgressed chromosomes are highly stable among the tissue types and during the cell cycle phases. High-throughput sequencing of Arabidopsis seedlings exposed to methyl jasmonic acid was performed by Zhang et al. [10] to identify differentially expressed circular RNAs. Based on their data, differentially expressed circular RNAs are involved in metabolic and developmental processes and are supposed to play important roles in methyl jasmonic acid-mediated signaling. Boudichevskaia et al. [11] dealt with the characterization of the role of Arabidopsis KNL2 (kinetochore null 2), which is important for deposition of CENH3 at centromeric regions. Transcriptomic analysis of mutant plants reveals that the *KNL2* gene loss of function affects processes of cell cycle regulation, transcription, development, and DNA repair. In the review article by R.M.S. et al. [12], the impact of redox components on activities of important epigenetic regulators was described. Authors provide an integrated view on the roles of oxidants (reactive oxygen species and nitric oxide) and antioxidants (pyridine nucleotides and glutathione) in the modulation of DNA methylation and histone modifications in plants.

Together, this collection offers diverse insights into the current plant epigenetics to allow readers to update their knowledge on the described phenomena and mechanisms in this highly complex and quickly evolving field. 

## Figures and Tables

**Table 1:** Contributors to the special issue “Chromatin, Epigenetics and Plant Physiology”.

Authors	Title	Type
Wang et al. [3]	Roles of the INO80 and SWR1 Chromatin Remodeling Complexes in Plants	Review
Guo et al. [4]	Mutations in the Rice OsCHR4 Gene, Encoding a CHD3 Family Chromatin Remodeler, Induce Narrow and Rolled Leaves with Increased Cuticular Wax	Original Research
Zhao et al. [5]	Identification and Characterization of Tomato SWI3-Like Proteins: Overexpression of SlSWIC Increases the Leaf Size in Transgenic Arabidopsis	Original Research
Gratkowska-Zmuda et al. [6]	The SWI/SNF ATP-Dependent Chromatin Remodeling Complex in Arabidopsis Responds to Environmental Changes in Temperature-Dependent Manner	Original Research
Krispil et al. [7]	The Position and Complex Genomic Architecture of Plant T-DNA Insertions Revealed by 4SEE	Original Research
Lochmanová et al. [8]	Different Modes of Action of Genetic and Chemical Downregulation of Histone Deacetylases with Respect to Plant Development and Histone Modifications	Original Research
Koláčková et al. [9]	Nuclear Disposition of Alien Chromosome Introgressions into Wheat and Rye Using 3D-FISH	Original Research
Zhang et al. [10]	Identification and Characterization of circRNAs Responsive to Methyl Jasmonate in Arabidopsis thaliana	Original Research
Boudichevskaia et al. [11]	Depletion of KNL2 Results in Altered Expression of Genes Involved in Regulation of the Cell Cycle, Transcription, and Development in Arabidopsis	Original Research
R.M.S. et al. [12]	Redox Components: Key-Regulators of Epigenetic Modifications in Plants	Review
